# Ethephon Poisoning: Clinical Characteristics and Outcomes

**DOI:** 10.3390/toxics13020115

**Published:** 2025-01-31

**Authors:** Satariya Trakulsrichai, Kanokrat Chuayaupakarn, Phantakan Tansuwannarat, Panee Rittilert, Achara Tongpoo, Charuwan Sriapha, Winai Wananukul

**Affiliations:** 1 Department of Emergency Medicine, Faculty of Medicine Ramathibodi Hospital, Mahidol University, Bangkok 10400, Thailand; kanokrat.mc3@gmail.com; 2 Ramathibodi Poison Center, Faculty of Medicine Ramathibodi Hospital, Mahidol University, Bangkok 10400, Thailand; phantakan.tans@gmail.com (P.T.); rittilert@gmail.com (P.R.); achara.ton@mahidol.ac.th (A.T.); charuwan.sri@mahidol.edu (C.S.); winai.wan@mahidol.edu (W.W.); 3 Emergency Department, Maharaj Nakhon Si Thammarat Hospital, Nakhon Si Thammarat 80000, Thailand; 4 Chakri Naruebodindra Medical Institute, Faculty of Medicine Ramathibodi Hospital, Mahidol University, Samut Prakan 10540, Thailand; 5 Department of Medicine, Faculty of Medicine Ramathibodi Hospital, Mahidol University, Bangkok 10400, Thailand

**Keywords:** 2-chloroethylphosphonic acid, plant growth regulator, plant hormone, mortality rate

## Abstract

Ethephon (2-chloroethylphosphonic acid) is a generally used plant growth regulator, but the data on its toxic effects, especially in humans, are very limited. This study was conducted to describe the clinical characteristics, management, and outcomes of patients exposed to products containing ethephon. We performed an 8-year retrospective study using data from the Ramathibodi Poison Center database (2013–2020), which included 252 patients. Most patients were male, with a median age of 32 years. Almost all patients were exposed through ingestion, mainly in unintentional circumstances. The clinical presentations included local effects, gastrointestinal (GI), neurological, and respiratory symptoms. Some patients required hospital admission; specifically, seven patients received inotropic drugs, and six were intubated with ventilator support. Most patients had either no or only minor clinical effects. However, six patients experienced moderate/severe effects, and two patients died. Age, intentional exposure, and the presence of initial neurological symptoms could prognosticate moderate to fatal outcomes. In conclusion, exposure to ethephon predominantly resulted in no or minor effects, and GI symptoms were the most common clinical manifestation. The cholinergic toxic syndrome was not frequently observed. The mortality rate was very low. Patients presenting with factors associated with worse outcomes should be monitored closely for clinical deterioration and appropriately managed.

## 1. Introduction

Ethephon (2-chloroethylphosphonic acid) is a commonly used plant growth regulator to promote fruit ripening, abscission, flower induction, and other responses [1,2]. After being metabolized by the plant, it is converted into ethylene, a potent regulator of plant growth and maturity. It is frequently used in agriculture for plenty of crops, including wheat, coffee, rice, and cotton [1].

Ethephon is a cholinesterase or acetylcholinesterase (AChE) inhibitor. Previous animal and human studies demonstrate that the plasma and erythrocyte cholinesterase were inhibited at all doses [2,3,4,5].

Symptoms indicating inhibition of cholinesterase activity were described in animals and human volunteers [5,6]. The reported toxic effects also include corrosive effects following acute oral, inhalation, and dermal exposure [2,3]. Based on studies conducted on animals [7,8,9,10,11], repeated exposure to this substance has been found to cause hepatotoxicity and nephrotoxicity, as well as potentially harmful effects on reproduction and fetal development. Ethephon is widely used in many countries [12]. It is marketed and available in Thailand as well, especially as a soluble concentrate (SL) formulation. The clinical data and studies regarding ethephon poisoning in humans are currently very limited.

Numerous patients exposed to ethephon-containing products in Thailand have been referred to the Ramathibodi Poison Center (RPC) for consultations. Therefore, this study was carried out to describe the clinical characteristics, treatment, and outcomes of patients with a history of acute exposure to ethephon-containing products by analyzing data from the RPC database.

## 2. Materials and Methods

### 2.1. Study Design

We performed an 8-year retrospective cross-sectional study by using data from the RPC database (RPC Toxic Exposure Surveillance System) from January 2013 to December 2020. The primary objective of this study was to outline the clinical characteristics and outcomes of patients exposed to products containing ethephon. The secondary objective was to indicate the prognostic factors contributing to moderate to fatal outcomes.

### 2.2. Study Setting and Population

RPC is a tertiary teaching hospital poison center that provides consultations via telephone for healthcare providers and the public 24 h a day with approximately 15,000–29,000 calls/year during the study period. Follow-up calls are arranged to evaluate the patient’s clinical progress and outcomes and provide further treatment recommendations if the patient’s condition changes. All data were recorded in the RPC database, and the team of senior information scientists and clinical toxicologists finally reviewed and validated the data.

We included the patients who were exposed to products containing ethephon through every route and were referred to the RPC for consultations in our study. The poisoning from ethephon-containing products was diagnosed based on a history of exposure to the products. The exposure was indicated by identifying the ingredients listed on the bottles if the container was brought to the hospital or by obtaining the brand name and detailed list of ingredients from the patients. The patients who co-ingested herbs, illicit drugs, pesticides, or other chemicals were excluded. We also excluded patients who overdosed on pharmaceutical drugs.

We collected patients’ data, including demographic data, medical history, clinical information, laboratory results, treatments, clinical course during hospitalization, and clinical outcomes.

### 2.3. Definitions

The estimated amount of ingestion is as follows: a teaspoon of the product equals 5 milliliters (mL), a mouthful is 25 mL (in adults), and a cup/glass is 250 mL [13,14].

This study defines hypotension and fever/high temperature as a systolic blood pressure < 90 mmHg and body temperature > 37.7 °C [15,16]. Abnormal heart rates, defined as bradycardia and tachycardia, occur when heart rates are below 60 and above 100 beats per minute, respectively [17]. The normal vital signs for pediatric patients were determined based on the normal range for their age [18].

Acute kidney injury was noted following the Kidney Disease: Improving Global Outcomes (KDIGO) Clinical Practice Guideline for Acute Kidney Injury [19]. Patients without preexisting conditions were presumed to have normal kidney function prior to exposure. Hypernatremia was defined as a serum sodium level > 145 mEq/L, while hyponatremia was defined as a serum sodium level < 135 mEq/L [20]. The definitions for hyperkalemia and hypokalemia were serum potassium levels > 5.0 mEq/L and <3.5 mEq/L, respectively [20]. Metabolic acidosis was defined as arterial pH < 7.40 and serum bicarbonate < 24 mEq/L [21], or as documented in a patient’s records. A high anion gap is defined as being >12 mEq/L [21]. The Glasgow Coma Scale (GCS) is scored between 3 and 15 [22]. The definitions and terms for grading the severity and effects of acute poisoning that are used in the RPC database were derived from the Poisoning Severity Score [23,24].

### 2.4. Statistical Analysis

The study data were collected and statistically analyzed using Microsoft Office 2019 (version 17, Redmond, WA, USA) and STATA software (StataCorp, College Station, TX, USA), respectively. Continuous data are reported as the mean and standard deviation when they are normally distributed; otherwise, the median with minimum and maximum range is presented. For categorical data, the frequency and percentage were reported. For normally distributed continuous data, a Student’s *t*-test was used for comparisons between groups, while a Mann–Whitney U test was employed for non-normally distributed data. Categorical data were analyzed to identify differences between groups using either a chi-square test or Fisher’s exact test. Logistic regression was used for univariate analysis, and the forward selection method was used for multivariate analysis. A *p*-value of less than 0.05 was considered statistically significant.

## 3. Results

There were a total of 258 consultations regarding exposure to ethephon-containing products. After the exclusion of patients with co-ingestion of other substances, 252 patients were included in our study. Table 1 presents the clinical characteristics of all the patients. A total of 166 patients (65.9%) were male; the median age was 32 years (range, 0.9–86). Patients were from every region in Thailand, with the most commonly reported cases arising from the eastern region (36.1%). Almost all patients were exposed through ingestion (98.8%), while three were exposed through inhalation. No patients were exposed via dermal contact. Accidental exposure accounted for 83.3% (*n* = 210), while intentional exposure or suicidal attempts were delineated in 16.7% (*n* = 42). The median duration from exposure to hospital visits was 60 min. The median amount ingested was 25 mL.

The clinical effects at presentation are summarized in Table 2. The clinical presentations mostly were gastrointestinal (GI) symptoms (55.6%). A total of 54 patients (21.4%) had no obvious symptoms. At presentation, 35 patients (13.9%) had tachycardia, 8 patients (3.2%) had bradycardia, and 2 patients (0.8%) had a high temperature. One patient (0.4%) had hypotension. Five patients (2%) had a Glasgow Coma Scale (GCS) score of less than 15 (range 3–14).

The results of the initial laboratory tests for electrolyte levels and kidney function are presented in Table 3. Most patients had normal results of initial laboratory tests. Two patients had hypernatremia (serum sodium of 148 and 149 mEq/L), and one patient had hyponatremia (serum sodium of 124 mEq/L). A total of 16 patients experienced hypokalemia (with a serum potassium range of 2.7–3.4 mEq/L), and 2 patients had hyperkalemia (serum potassium of 5.3 and 6.1 mEq/L). A high anion gap metabolic acidosis was reported in two patients with anion gaps of 25 and 33 mEq/L.

A total of 244 patients (96.8%) had no or minor effects. Some 108 patients (42.9%) were admitted to hospitals, with a median length of hospital stay of 2 days, ranging from 1 to 14 days. The lengths of hospital stay of 17 admitted patients were not documented in our database for the reason that they recovered from poisoning and then were not followed up by the RPC until their discharge. Two patients developed AKI during hospitalization. Supportive care was the main treatment for almost all of patients, and they were finally discharged.

Eight patients (3.2%) had moderate, severe and fatal outcomes (Table 4). Seven of the patients were male, and six of them intentionally ingested ethephon. The maximum amount of ingestion was 300 mL (Patient 7). Six patients were admitted to the intensive care unit. Six patients underwent an endotracheal intubation with mechanical ventilator support. Seven patients received inotropes. The most common complication during hospitalization was pneumonia, noted in four patients. Two patients died, resulting in a mortality rate of 0.8%.

The dead patients are summarized as follows (Table 4).

The first dead case (Patient 1, Table 4) was a 40-year-old male with multiple underlying diseases, including alcohol dependence. He intentionally ingested a product containing ethephon in an unknown amount. At the scene, he had hypersalivation, nausea, and vomiting. One hour after exposure, he arrived at the emergency department with altered consciousness, a low GCS score (E2V2M5), and sinus bradycardia. The initial venous blood gas (VBG) showed acute respiratory acidosis. Approximately 1 h later, his electrocardiogram demonstrated pulseless electrical activity. Subsequently, cardiopulmonary resuscitation (CPR), intravenous (IV) adrenaline, and endotracheal intubation were performed. Hypersecretion was observed, and IV atropine was administered to treat this condition. Four hours later, he regained consciousness and received IV thiamine, dextrose, and norepinephrine. One day after admission, he developed pneumonia and upper GI bleeding. On the second day of hospitalization, he experienced hypotension and eventually died.

The second dead case (Patient 2, Table 4) was an 80-year-old male with a history of hypertension and coronary artery disease. He accidentally ingested 25 mL of ethephon. Thirty minutes after ingestion, he had no apparent symptoms. Hypokalemia (serum potassium of 2.66 mEq/L) was initially observed. He was treated with an IV antiemetic drug and IV fluids with a potassium chloride supplement. He developed tachypnea, fever (body temperature 38.5 °C), and crepitation of both lungs. He was then intubated with ventilator support. He experienced diarrhea several times (>20 times), sinus tachycardia (heart rate 166 bpm), depressed consciousness (GCS score: E4VTM5), and oliguria. His treatment included comprehensive supportive care and consulting a gastroenterologist for severe diarrhea evaluation. On the sixth day of admission, his chest X-ray showed right lung infiltration, and VBG showed respiratory and metabolic acidosis. He was diagnosed with severe pneumonia with septic shock. IV meropenem and norepinephrine were administered. After 10 days of admission, he died from complications.

We compared the clinical manifestations between patients with no/minor and moderate/severe/fatal outcomes to analyze the factors associated with moderate, severe, and fatal outcomes/severities. The amount of ingestion, intentional exposure, neurological symptoms, and GCS score at presentation <15 showed statistically significant differences (Table 5).

Table 6 presents the univariate and multivariate analysis of factors associated with moderate, severe, and fatal outcomes/severities. In the multivariate analysis, age, intentional exposure, and the presence of neurological symptoms at presentation were associated with worse outcomes.

## 4. Discussion

In this study, we examined patients who had been exposed to ethephon to analyze their clinical characteristics and outcomes. According to the limited data on ethephon poisoning in humans within the literature in English, this study, which described 252 patients, is one of the largest conducted regarding ethephon poisoning. The findings of the study provide significant data that expand the existing knowledge on this poisoning. The importance of this study on the toxicity of ethephon is emphasized. The majority of the patients were exposed orally, mostly through accidental circumstances. Our findings suggest that the containers of these products might be easily accessible or that ethephon may be stored in other food containers, increasing the risk of accidental ingestion. In addition, unclear or absent labeling on the product bottles may have contributed to unawareness about their contents. This finding suggested that implementing safety measures, such as clear product labeling or appropriate containers, could be a strategy to prevent this poisoning.

Most patients presented with no apparent symptoms or mild clinical effects, with normal vital signs at presentation. The median lethal dose (LD50) of ethephon is >4000 mg/kg. The WHO recommended classification of pesticides by hazard categorizes ethephon as classification III (Slightly hazardous) [25]. Therefore, our findings supported this classification.

Local irritation was also observed in our patients, consistent with the findings of the previous study [2,3]. GI symptoms were the most common clinical finding in our study. Neurological symptoms, especially depressed consciousness, were occasionally reported. Although ethephon is an AChE inhibitor, prominent symptoms of cholinergic toxic syndrome were not commonly reported in our patients. Hypersecretion and diarrhea were observed in some patients. However, plasma and erythrocyte cholinesterase levels were not evaluated in these patients. Previous findings in animal studies involving repeated exposure revealed liver toxicity [7,8]. The patients in our study did not exhibit hepatotoxicity. Therefore, in acute poisoning, liver toxicity is not a clinical feature. The cornerstone of management was supportive care.

Six patients with worse outcomes (moderate to fatal final severities) were exposed through intentional ingestion. Intentional circumstances may lead to greater exposure than unintentional ones and contribute to significant clinical effects. In our study, two patients died. Although the mortality rate was very low, patients experiencing this poisoning could still be at risk of death. One patient died rapidly after intentional exposure, developing consciousness depression and complications. The other one accidentally ingested and died from complications of pneumonia 10 days later.

According to the multivariate analyses, we found that age, intentional exposure, and having neurological symptoms at presentation were prognostic factors associated with moderate, severe, or fatal outcomes. Therefore, elderly patients with intentional exposure or having neurological symptoms should be closely and carefully observed, monitored, and well managed. Adequate supportive care and appropriate management of laboratory abnormalities or complications during admission are essential for all patients.

The current study has several limitations, as follows. The data may not reflect the true incidences of poisoning and mortality in Thailand, as reporting exposure to ethephon-containing products to the RPC is not mandatory. The retrospective study design may have led to incomplete or unclear data. The medical histories were collected from patients or their relatives, who reported to the medical staff. These histories may not have been clearly or entirely documented. The diagnosis was primarily based on the patient’s exposure history, without specific laboratory confirmation of ethephon exposure.

## 5. Conclusions

Ethephon poisoning commonly results in no effects or only mild symptoms. GI symptoms were the predominant clinical manifestations. It is important to note that cholinergic toxic syndrome is not a typical clinical feature associated with this type of poisoning. The mortality rate was very low. Supportive care may be considered as the main treatment. Age, intentional exposure, and neurological symptoms at presentation were prognostic factors for worse outcomes. Consequently, patients with these factors should be closely observed and monitored for clinical deterioration.

## Figures and Tables

**Table 1:** Clinical characteristics of all patients.

Clinical Characteristics (Number of Patients with Data Available)	*n* (%)
Sex	
Male	166 (65.9)
Female	86 (34.1)
Age (years), median (min–max)	32 (0.9–86)
Region	
Central *	31 (12.3)
North	8 (3.2)
North-East	44 (17.5)
East	91 (36.1)
West	19 (7.5)
South	59 (23.4)
Duration from exposure to hospital visits (minutes), median (min-max) (248 patients)	60 (5–8640)
Amount of ingestion (milliliters), median (min-max) (219 patients)	25 (0.2–500)

* Including Bangkok.

**Table 2:** Clinical presentations of all patients.

Clinical Presentations *	*n* (%)
No apparent symptoms	54 (21.4)
Local symptoms	
-Oral mucosa irritation	89 (35.3)
-Dermal or ocular irritation	2 (0.8)
Gastrointestinal (GI) symptoms **	140 (55.6)
Neurologic symptoms ***	23 (9.1)
Respiratory symptoms ****	4 (1.6)

* Some patients presented with > 1 symptom. ** Nausea/vomiting (110 patients), abdominal pain (61 patients), hypersalivation (13 patients), diarrhea (9 patients), GI bleeding (5 patients), and constipation (2 patients). *** Dizziness (10 patients), drowsiness (7 patients), and headache (2 patients). **** Dyspnea (4 patients) and chest discomfort (1 patient).

**Table 3:** Results of initial laboratory tests for electrolyte levels and kidney function of all patients.

Laboratory Results (Number of Patients with Data Available)	Value
Serum sodium (mEq/L), mean ± SD (50 patients)	139.1 ± 3.8
Serum potassium (mEq/L), mean ± SD (56 patients)	3.8 ± 0.6
Serum chloride (mEq/L), mean ± SD (50 patients)	104.7 ± 4.3
Serum bicarbonate (mEq/L), mean ± SD (50 patients)	22.1 ± 5.0
Serum blood urea nitrogen (mg/dL), mean ± SD (39 patients)	11.7 ± 3.5
Serum creatinine (mg/dL), mean ± SD (39 patients)	0.9 ± 0.3

**Table 4:** Clinical characteristics and laboratory findings of patients with moderate, severe, and fatal outcomes.

Clinical Characteristics	Patient 1	Patient 2	Patient 3	Patient 4	Patient 5	Patient 6	Patient 7	Patient 8
**Patient characteristics**								
Sex/Age (years)	Male/40	Male/80	Male/55	Male/52	Male/26	Male/40	Male/41	Female/28
Underlying diseases	AD, HIV infection, MDD	HT, CAD	AD	No	No	AD	No	No
Duration after exposure to hospital visits (minutes)	60	30	30	60	60	60	60	120
Circumstances	Intentional	Accidental	Intentional	Intentional	Intentional	Accidental	Intentional	Intentional
Amount of ingestion (mL)	N/A	25	N/A	25	N/A	25	300	75
**Clinical manifestations at presentation**								
**Local effects** (yes/no)								
Oral mucosa irritation	No	Yes	No	Yes	No	No	No	No
Salivation	Yes	No	No	No	Yes	No	Yes	Yes
GI symptoms (yes/no)	Yes	Yes	Yes	No	No	Yes	No	Yes
Neurologic symptoms (yes/no)	Yes	No	No	No	Yes (seizure)	Yes (tremor)	Yes	Yes
Respiratory symptoms (yes/no)	Yes	No	Yes	No	No	No	No	Yes
**Systemic effects at presentation**								
**Vital signs:**								
BT (°C), HR (/minute), RR (/minute), BP (mmHg)	N/A 60 18 112/53	N/A N/A N/A N/A	N/A 70 N/A 70/30	36.7 98 20 160/96	36.6 78 18 160/90	37.2 98 20 138/82	37.5 108 20 137/87	N/A 40 N/A N/A
GCS	9	15	15	15	7	15	12	6
Metabolic acidosis (yes/no) Anion gap (mEq/L)	Yes 33	No 5	Yes 25	No 4	No 10	Yes 22	No 9	N/A N/A
Acute kidney injury (yes/no) Serum BUN, Creatinine (mg/dL)	No 8, 1.1	Yes N/A	No N/A	No 13, 0.87	No 10, 0.8	Yes 7, 1.8	No N/A, 0.89	No N/A
**Treatment** (yes/no)								
Oxygen therapy	Yes	Yes	Yes	Yes	No	Yes	Yes	Yes
Endotracheal intubation Administration of inotropes	Yes Yes	Yes Yes	Yes Yes	Yes No	No Yes	Yes Yes	No Yes	Yes Yes
**Hospital clinical course**								
**Complication during hospitalization** (yes/no)								
Pneumonia	Yes	Yes	No	No	No	Yes	No	Yes
Acute kidney injury	Yes	Yes	Yes	No	No	Yes	No	Yes
Hospital stays (days)	2	10	N/A	N/A	2	N/A	6	14
Outcomes	Death	Death	Severe	Moderate	Moderate	Severe	Moderate	Moderate

**Abbreviations:** AD, Alcohol dependence; MDD, major depressive disorder; HIV, human immunodeficiency virus; HT, hypertension; CAD, coronary artery disease; N/A, not applicable; mL, milliliters; BUN, blood urea nitrogen.

**Table 5:** Comparison of clinical manifestations between patients who had no/minor and moderate to fatal outcomes.

Clinical Manifestations (Number of Patients with Data Available)	No/Minor Outcomes (*n* = 244)	Moderate/Severe/Fatal Outcomes (*n* = 8)	*p*-Value
Sex: number of male: female (252 patients)	159:85	7:1	0.271
Age (years), median (min–max) (252 patients)	31 (0.92–86)	40.5 (26–80)	0.387
Amount of ingestion (milliliters), median (min–max) (*n* = 219)	25 (0–500)	50 (25–300)	0.035
Number of unintentional: intentional exposure (252 patients)	208:36	2:6	<0.001
Time to hospital (minutes)	60 (5–8640)	60 (30–60)	1.000
Clinical presentations			
Gastrointestinal symptoms Neurologic symptoms Respiratory symptoms Glasgow coma scale score < 15	134 (54.9) 18 (7.4) 3 (1.2) 3 (1.2)	6 (75.0) 5 (62.5) 1 (12.5) 2 (25.0)	0.306 <0.010 0.122 0.008

**Table 6:** Univariate and multivariate analyses of factors associated with moderate, severe, and fatal outcomes.

Factors	Univariate Analysis			Multivariate Analysis		
	Odds Ratio (OR)	95%CI	*p*-Value	Odds Ratio (OR)	95%CI	*p*-Value
Sex	3.742	0.453–30.921	0.221	-	-	-
Age (years)	1.032	0.997–1.068	0.076	1.107	1.024–1.197	0.011
Amount of ingestion (milliliters)	1.006	0.999–1.012	0.093	-	-	-
Number of unintentional: intentional exposures	17.333	3.366–89.262	0.001	45.557	3.650–568.591	0.003
Time to hospital	0.995	0.983–1.007	0.392	-	-	-
Clinical presentations						
Gastrointestinal symptoms	2.463	0.487–12.445	0.276	-	-	-
Neurologic symptoms	20.926	4.624–94.697	<0.001	57.377	5.724–575.120	0.001
Respiratory symptoms	11.476	1.057–124.580	0.045	-	-	-
Glasgow coma scale score < 15	26.778	3.757–190.871	0.001	-	-	-

## Data Availability

The data in this article were presented at the North American Congress of Clinical Toxicology (NACCT) 2023, Montreal, Quebec, Canada, 29 September–1 October 2024, as a poster presentation with interim findings. The data are inaccessible to the public due to patient privacy concerns, but they can be obtained from the corresponding author upon reasonable request.
